# A Highly Selective In Vitro JNK3 Inhibitor, FMU200, Restores Mitochondrial Membrane Potential and Reduces Oxidative Stress and Apoptosis in SH-SY5Y Cells

**DOI:** 10.3390/ijms22073701

**Published:** 2021-04-02

**Authors:** Stephanie Cristine Hepp Rehfeldt, Stefan Laufer, Márcia Inês Goettert

**Affiliations:** 1 Graduate Program in Biotechnology, University of Vale do Taquari (Univates), Lajeado, RS 95914-014, Brazil; srehfeldt@universo.univates.br; 2 Department of Pharmaceutical and Medicinal Chemistry, Institute of Pharmacy, Eberhard Karls Universität Tübingen, D-72076 Tübingen, Germany; 3 Tübingen Center for Academic Drug Discovery (TüCAD2), D-72076 Tübingen, Germany

**Keywords:** 6-hydroxydopamine, hydrogen peroxide, apoptosis, mitochondrial membrane potential, neurodegenerative diseases, oxidative stress

## Abstract

Current treatments for neurodegenerative diseases (ND) are symptomatic and do not affect disease progression. Slowing this progression remains a crucial unmet need for patients and their families. c-Jun N-terminal kinase 3 (JNK3) are related to several ND hallmarks including apoptosis, oxidative stress, excitotoxicity, mitochondrial dysfunction, and neuroinflammation. JNK inhibitors can play an important role in addressing neuroprotection. This research aims to evaluate the neuroprotective, anti-inflammatory, and antioxidant effects of a synthetic compound (FMU200) with known JNK3 inhibitory activity in SH-SY5Y and RAW264.7 cell lines. SH-SY5Y cells were pretreated with FMU200 and cell damage was induced by 6-hydroxydopamine (6-OHDA) or hydrogen peroxide (H_2_O_2_). Cell viability and neuroprotective effect were assessed with an MTT assay. Flow cytometric analysis was performed to evaluate cell apoptosis. The H_2_O_2_-induced reactive oxygen species (ROS) generation and mitochondrial membrane potential (ΔΨm) were evaluated by DCFDA and JC-1 assays, respectively. The anti-inflammatory effect was determined in LPS-induced RAW264.7 cells by ELISA assay. In undifferentiated SH-SY5Y cells, FMU200 decreased neurotoxicity induced by 6-OHDA in approximately 20%. In RA-differentiated cells, FMU200 diminished cell death in approximately 40% and 90% after 24 and 48 h treatment, respectively. FMU200 reduced both early and late apoptotic cells, decreased ROS levels, restored mitochondrial membrane potential, and downregulated JNK phosphorylation after H_2_O_2_ exposure. In LPS-stimulated RAW264.7 cells, FMU200 reduced TNF-α levels after a 3 h treatment. FMU200 protects neuroblastoma SH-SY5Y cells against 6-OHDA- and H_2_O_2_-induced apoptosis, which may result from suppressing the JNK pathways. Our findings show that FMU200 can be a useful candidate for the treatment of neurodegenerative disorders.

## 1. Introduction

**Figure 1:**
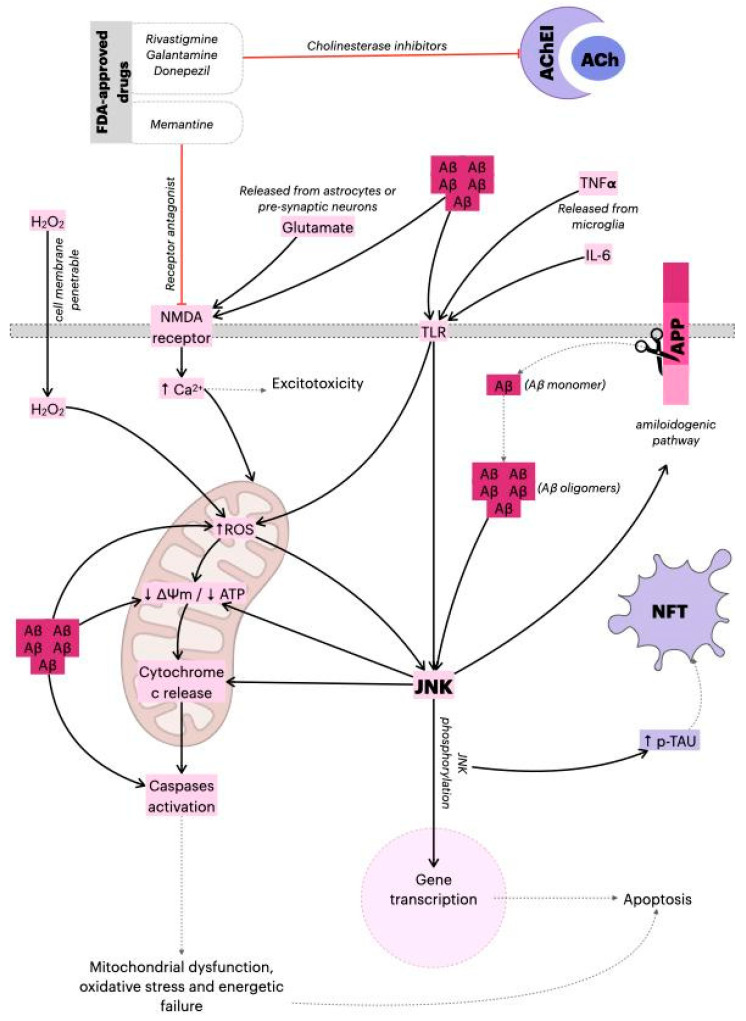
Mechanism of action of FDA-approved drugs and the role of JNK in AD. The Aβ monomers are generated by amyloid precursor protein (APP) cleavage, and subsequently released from neurons. The monomers sequentially assemble into Aβ oligomer aggregates, and ultimately into amyloid plaques. The Aβ oligomers can alter mitochondria function and induce ROS production, dissipation of ΔΨm, caspase-3 activation, and ATP reduction. Additionally, Aβ oligomers activate the JNK pathway, thereby aggravating synaptic dysfunction. As a result, phosphorylated JNK induces dissipation of ΔΨm, cytochrome c release, and the activation of transcription factors such as AP-1 or c-Jun, which leads to apoptosis. However, JNK directly phosphorylates Tau and induces the amyloidogenic pathway, contributing to the formation of neurofibrillary tangles (NFTs) and amyloid plaques, respectively, causing the gradual loss of cholinergic neurons in Alzheimer’s disease (AD). The main pharmacological actions of donepezil, galantamine and memantine (acetylcholinesterase/cholinesterase inhibitors (AChEI)) are believed to occur as the result of acetylcholinesterase activity inhibition. By blocking breakdown of ACh, the cholinergic transmission is enhanced, and the symptoms of AD are relieved. Additionally, in response to Aβ aggregation, microglia release pro-inflammatory cytokines (TNF-α, IL-6) that activate astrocytes and induce apoptotic signals in neurons via TLR-JNK activation. The excessive activation of the NMDA receptor by glutamate results in excitotoxicity and Ca^2+^-dependent cell death. Memantine (N-methyl-D-aspartic acid (NMDA) receptor antagonist) inhibits calcium influx into cells that is normally caused by chronic NMDA receptor activation by glutamate. This leads to the improvement of Alzheimer’s dementia symptoms, demonstrated by increased cognition and other beneficial central nervous system effects [22,23,24]. ↓: represents decrease; ↑ represents increase.

In neurodegenerative disorders (ND), such as Alzheimer’s disease (AD), it is common that neurons start degenerating during a prolonged preclinical period, where individuals are by definition asymptomatic and cognitively normal [1]. In recent years, researchers have put more effort into understanding neurodegenerative diseases and investing in patient management based on molecular approaches with potential disease-specific and/or disease-modifying treatments specifically targeting neuroprotection. Neuroprotection can be characterized as a substantial and lasting slowdown in the disease’s progression associated with a delay in neuronal degeneration [2]. While a few drugs can improve the patient’s quality of life, there is neither a cure nor a disease-modifying drug for treating AD and the disease inevitably progresses, making AD fatal in all cases. According to the U.S. Food and Drug Administration (FDA) and the Alzheimer’s Association, there are only five FDA-approved drugs to manage AD nowadays: donepezil, galantamine, memantine, rivastigmine, and a combination of memantine and donepezil [3] (Figure 1). The limited number of drugs and the failure of several drugs/compounds in phase III clinical trials (focused primarily on the amyloid hypothesis) [4] indicates that new targets should be explored.

In this sense, it is known that cell perturbations provoked by β-amyloid peptides (Aβ), neurofibrillary tangles, and oxidative stress, for example, can culminate in the activation of mitogen-activated protein kinase (MAPK) pathways, such as the JNK (c-Jun N-terminal kinase) pathway, best known for its involvement in propagating pro-apoptotic signals via extrinsic and intrinsic pathways [5,6]. Studies on post-mortem brain samples have shown a greater expression of phosphorylated JNK3 in AD patients in addition to the presence of (Aβ) [7], while further studies have identified JNK3 to be highly expressed and activated in brain tissue and cerebrospinal fluid in patients with AD, in addition to being statistically correlated with the level of cognitive decline [8,9].

On the other hand, mitochondria are considered the major source of reactive oxygen species (ROS) in the cell and the accumulation of ROS-associated damage in DNA, proteins, and lipids, and may cause progressive cell dysfunctions and, in consequence, apoptosis. For this reason, authors have recognized mitochondria as a critical organelle for various pathological conditions and aging. It was also shown that mitochondrial JNK signaling can impact mitochondrial physiology [10] likewise, and the culmination of oxidative stress in the mitochondria is the dissipation of mitochondrial membrane potential (MMP) and subsequent release of cytochrome c [11,12]. Therefore, the inhibition of JNK3 has been explored as a possible therapeutic target.

Kinase inhibitors are not a novel type of treatment [13,14]. This issue has been widely discussed over the last two decades and a review conducted by Koch et al. in 2015 [15] acknowledged the need for JNK isoform-specific inhibitors. The authors gave a detailed description of the available inhibitors’ chemical characteristics, but also pointed out the complexity of developing new drugs. Since then, some inhibitors cited in the paper have advanced to clinical trials or showed promising results in recent animal model studies. However, other compounds did not advance as much, especially due to a lack of specificity. Recently, the role of JNK3 in Alzheimer’s disease was reviewed [16]. The authors also pointed to JNK3 inhibitors explored so far with a promising future as therapeutic proposals for AD.

Although JNK3 is present in specific tissues such as the brain, heart, placenta, lung, liver, skeletal muscle, kidney, pancreas, and testis, over 500 kinases were identified in humans [17]. The structure of many MAPKs is very similar. JNK3 shares 77% and 75% amino acid sequence identity with JNK2 and JNK1, respectively. The sequence identity at the ATP-binding pocket is 98% identical between all three JNK isoforms [18]. The selectivity of JNK3 inhibitors is often pointed out to be problematic. Even though many JNK3 inhibitors are cited in the literature, most of them display very weak JNK3 selectivity and/or cannot properly inhibit the phosphorylation of JNK3 substrates [15,16,19]. In this sense, cysteine-directed covalent inhibitors possess an ability to control kinase selectivity using both non-covalent and covalent recognition of the kinase and the ability to exhibit prolonged pharmacodynamics [20]. FMU200 is a tetrasubstituted imidazole that forms a covalent bond with JNK3 described by Muth et al., 2016. This compound binds to the Cys-154 of JNK3 and shows a picomolar inhibitory effect for JNK3 and a 120-fold preference compared to the IC50 value of p38α, for example (IC50 JNK3: 0.3 nM). Within the JNK group, at 0.1 µM, FMU200 inhibits JNK1 activity by 16%, JNK2 by 73%, and JNK3 by 80%; at 0.5 µM FMU200, the residual activity of JNK1, JNK2, and JNK3 are 16%, 5% and, 4%, respectively [21]. Based on this molecule’s interesting inhibitory profile, this study aimed to explore the neuroprotective, anti-inflammatory and antioxidant potential of FMU200 in an in vitro model of neurodegenerative disease.

## 2. Results

### 2.1. The Cytotoxic and Neuroprotective Effect of FMU200 in Undifferentiated and RA-Differentiated SH-SY5Y Cells

The pharmacological inhibition of the MAPK pathway is a common research method used to understand mechanisms of cellular functions and fundamental processes [25,26]. A number of pharmacological inhibitors have been developed to block MAPKs either directly or indirectly, by targeting upstream regulators. In this case, we included an p38 inhibitor (SB203580) [27] and a JNK inhibitor, SP600125 [28] in our analysis. According to previous studies, SP600125 (10 µM) was considered cytotoxic in different cell lines such as four different leukemia cell lines (U937, K562, HL60, and THP-1) [29], NphA2 cells [30], while SB203580 and SP600125 at 20 µM did not alter cell viability neither in MCF7 cells [31] nor in JEG-3 cells [32]. However, it is known that distinct cell lines respond differently to cytotoxic compounds. Among 1353 compounds tested in 13 different cell types, SH-SY5Y was classified as the second most sensitive cell line to compound-induced toxicity. Overall, SH-SY5Y cells were more sensitive to compound-induced toxicity than SK-N-SH cells, the parental cell line of the SH-SY5Y cells [33]. Since there are conflicting results whether SP600125 and SB203580 are cytotoxic or not, and due to the highly heterogeneous cellular response to both SP600125 and SB203580, we started our concentration screening with a 10 µM concentration for all three compounds (FMU200, SP600125, and SB203580).

**Figure 2:**
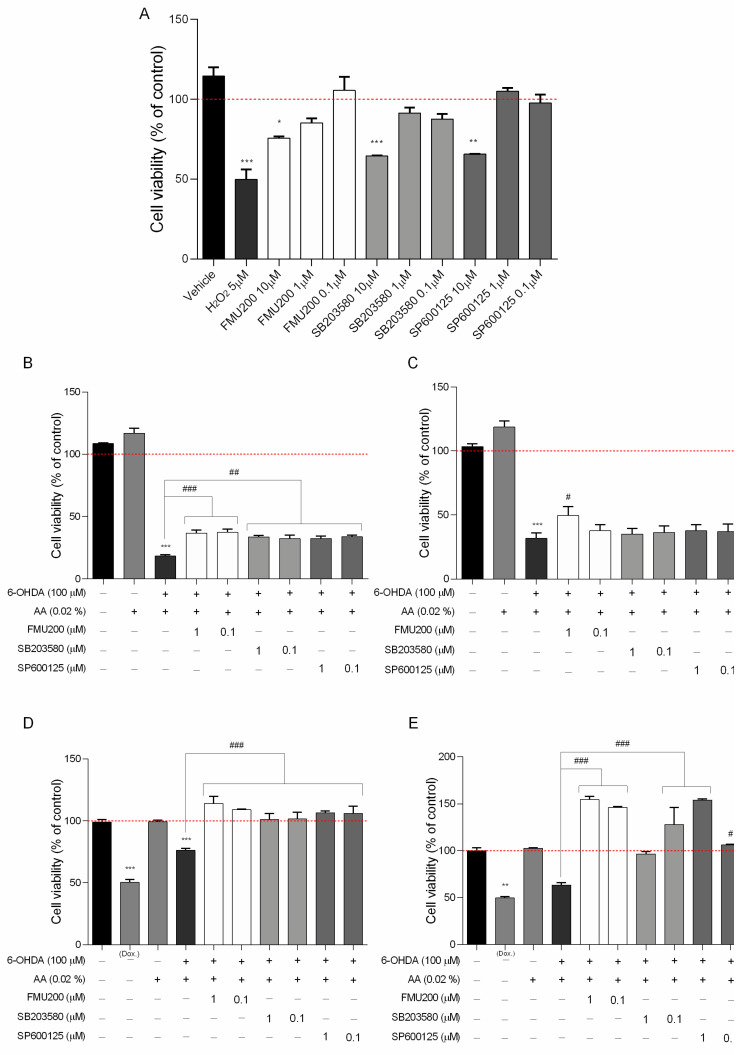
(**A**) Cytotoxicity induced by FMU200 on SH-SY5Y cell viability after a 24 h incubation period in SH-SY5Y cells (% of control); the neuroprotective effect of FMU200 against 6-OHDA (stabilized with 0.02% of ascorbic acid) induced neurotoxicity in undifferentiated SH-SY5Y cells and RA-differentiated SH-SY5Y cells. SH-SY5Y cells were pretreated with different concentrations of FMU200 for 1 h, prior to 6-OHDA exposure. Undifferentiated cells were incubated for (**B**) 24 h and (**C**) 48 h. Cells were differentiated in 10-μM retinoic acid (RA) and the effect was also evaluated in RA-differentiated cells after (**D**) 24 h and (**E**) 48 h. The results are the mean ± SEM of at least three experiments in triplicates. Statistical calculations were performed by ANOVA via the Tukey post hoc test. Statistical significance values were *** *p* < 0.001; ** *p* < 0.01; * *p* < 0.05 (vs. control); ### *p* < 0.001; ## *p* < 0.01; # *p* < 0.05 (vs. 6-OHDA). Negative control (untreated cells) was considered to be 100% viable and is represented by the red dashed line. Doxorubicin was used as positive control; DMSO 0.1% was used as vehicle.

In this case, the first set of analyses examined the impact of FMU200, SP600125 and SB203580 on cell viability. The results, as shown in Figure 2A, indicate that in SH-SY5Y cells, FMU200 at 10 µM reduced cell viability by 24.33% (*p* < 0.05). At 1 µM, FMU200 reduced cell viability by only 14.75% (*p* > 0.05). SB230580 at 10 µM reduced cell viability by 35.5% (*p* < 0.001), while SP600125 at 10μM reduced viability by 34.3% (*p* < 0.001). Since all three compounds (FMU200, SP600125, and SB203580) showed statistically significant cytotoxicity to SH-SY5Y cells at 10 µM, we did not use this concentration in further assays.

6-OHDA is a highly reactive and oxidizable molecule being rapidly and non-enzymatically oxidized by molecular oxygen to form hydrogen peroxide (H_2_O_2_). However, the presence of ascorbic acid (AA) decreased O_2_ consumption and H_2_O_2_ amount indicating that AA reduced the autoxidation rate of 6-OHDA [34]. Therefore, 6-OHDA was stabilized with 0.02% AA before being added to cells.

Undifferentiated and RA-differentiated SH-SY5Y neuroblastoma cells were pretreated with 1 and 0.1 μM of FMU200 for 1 h prior to 6-OHDA exposure and incubated for 24 or 48 h. Cell viability was examined with an MTT assay. For undifferentiated cells, 6-OHDA reduced cell viability by 81.65% (after 24 h) and by 68.42% (after 48 h) when compared to control (*p* < 0.001). Similarly, when compared to control, treatment with FMU200 at both concentrations also decreased cell viability. However, if compared to the 6-OHDA group, after 24 h, FMU200 at 1 µM and 0.1 µM increased cell viability by 18.29% (*p* < 0.001) and 18.9% (*p* < 0.001), respectively (Figure 2B). After 48 h treatment, FMU200 at 1 µM and 0.1 µM increased cell viability by 17.86% and 5.92%, respectively (Figure 2C).

For RA-differentiated cells, the cells were incubated with 10 μM RA for 10 days to induce neuronal differentiation before exposure to FMU200 and 6-OHDA. After 24 h, 6-OHDA decreased cell viability by 23.84% when compared to control (*p* < 0.001). Treatment with FMU200 at 1 µM and 0.1 µM did not show any significant difference when compared to control. On the other hand, FMU200 at 1 µM increased cell viability by 37.87%, while FMU200 at 0.1 µM increased cell viability by 32.83% (when compared to 6-OHDA, *p* < 0.001) (Figure 2D). After 48 h, 6-OHDA decreased cell viability by 36.54% (*p* < 0.05) when compared to control. When compared to 6-OHDA, treatment with FMU200 at 1 µM increased cell viability by 91.04% (*p* < 0.001), while FMU200 at 0.1 µM increased cell viability by 82.54% (*p* < 0.001) (Figure 2E).

### 2.2. Response to H_2_O_2_: Apoptosis, ROS Production and MMP

**Figure 3:**
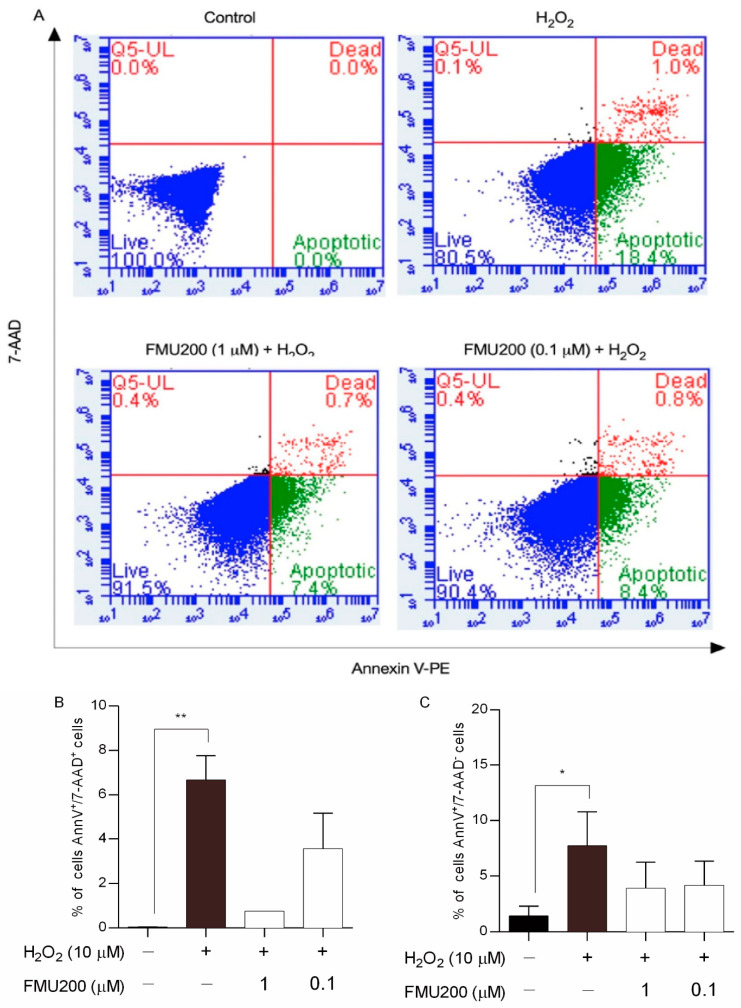
Effects of co-treatment with H_2_O_2_ and FMU200 after 6 h. (**A**) FL-2 (Annexin V-PE) vs. FL-3 (7-AAD) dot plots for untreated cells (control), cells treated with 10 µM H_2_O_2_ only, cells pretreated with 1 µM FMU200, and cells pretreated with 0.1 µM FMU200 for 1 h before H_2_O_2_ exposure; (**B**) percentage of dead cells (cells stained with both 7-AAD and Annexin V-PE); (**C**) percentage of early apoptotic cells (cells stained with Annexin V-PE only). The data are expressed as the means of three independent experiments together with the standard error of the mean (mean ± SEM). Statistical calculations were performed by ANOVA via the Tukey post hoc test. Statistical significance values were ** *p* < 0.01; * *p* < 0.05 (vs. control).

Apoptosis is an important event in neurodegeneration and flow cytometry-based apoptosis assays have proved to be especially useful as they offer an individual cell-based method of analysis. In this case, to further characterize H_2_O_2_-induced cell death, we performed flow cytometric analysis of SH-SY5Y cells using annexinV-PE and 7-AAD, which has been used to determine early apoptosis and necrosis/late apoptosis. Figure 3 shows the percentage of early apoptosis (FL2 positive/FL3 negative) and necrosis/late apoptosis (FL2 positive/FL3 positive) was increased in SH-SY5Y cells challenged with 10 µM of H_2_O_2_. Cells pretreated with FMU200 at 1 µM and 0.1 µM showed fewer annexinV-PE-positive/7-AAD-positive cells and fewer annexinV-PE-positive/7-AAD-negative cells.

**Figure 4:**
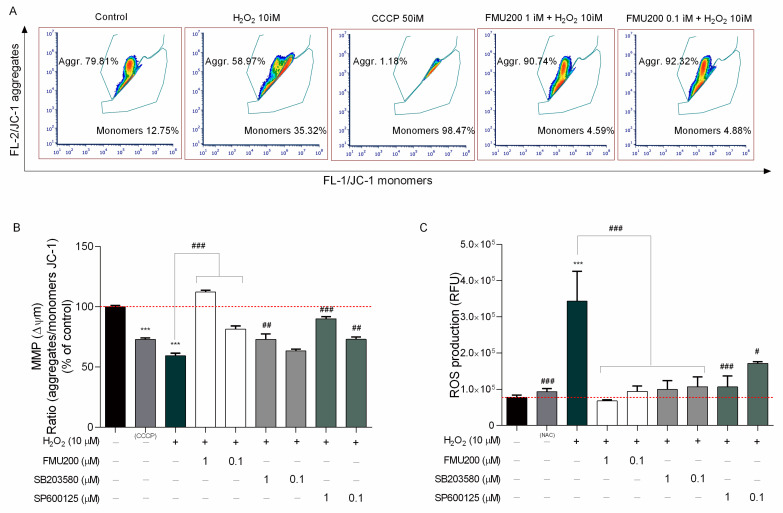
(**A**) Representative flow cytometric analysis showing that FMU200 inhibited H_2_O_2_-induced ΔΨm depolarization monitored by JC-1 dye; (**B**) changes of mitochondria stained with JC-1 were also detected using a fluorescence microplate reader. The ratio of red fluorescence to green fluorescence of the control was defined as 100%; (**C**) intracellular ROS levels were measured by DCF-DA staining in SH-SY5Y cells after H_2_O_2._-induced damage. Data are expressed as relative fluorescence unit (RFU) per cell. The data are expressed as the means of three independent experiments together with the standard error of the mean (mean ± SEM). Statistical calculations were performed by ANOVA via the Tukey post hoc test. Statistical significance values were *** *p* < 0.001 (vs. control); ### *p* < 0.001; ## *p* < 0.01; # *p* < 0.05 (vs. H_2_O_2_).

Reactive oxygen species (ROS) are involved in both physiological and pathological processes and mitochondria are widely accepted as the major site for ROS formation. The mitochondrial membrane potential (ΔΨm) reflects the functional metabolic status of mitochondria. Mitochondrial dysfunction with a loss of mitochondrial membrane potential (MMP) is also a critical event in neuronal degeneration. JC-1 dye can selectively enter mitochondria and reversibly change color from red to green as the membrane potential decreases. In flow cytometric analysis of JC-1 fluorescence, healthy cells with high mitochondrial ΔΨm, JC-1 spontaneously forms complexes known as J-aggregates with intense red fluorescence. On the other hand, in apoptotic or unhealthy cells with low ΔΨm, JC-1 remains in the monomeric form, which shows only green fluorescence (Figure 4A).

However, since SH-SY5Y cells are required to be in suspension, we also determined the ΔΨm and ROS production with fluorescence microplate reader since trypsinization may induce oxidative stress [35,36]. In this case, in order to determine if the inhibitor FMU200 had an impact on JNK-mediated physiological responses, such as loss of MMP and ROS generation induced by H_2_O_2_, cells were treated as previously described and incubated with JC-1 dye to monitor the MMP. The samples were analyzed by detecting the red fluorescence and green fluorescence ratio. Exposure of SH-SY5Y cells to H_2_O_2_ decreased the ratio of red fluorescence/green fluorescence by 40.57% compared with control (*p* < 0.001). However, co-treatment with FMU200 at 1 µM and 0.1 µM increased MMP by 52.79% and 22.08%, respectively (*p* < 0.001) (Figure 4B).

The 2′-7′-Dichlorodihydrofluorescein diacetate (DCFH-DA) is one of the most widely used techniques for directly measuring the redox state of a cell. In order to determine if FMU200 could protect cells against oxidative stress caused by H_2_O_2_, cellular ROS formation was quantified by the DCFDA assay. Cells were pretreated with FMU200 at 1 µM and 0.1 µM prior to H_2_O_2_ exposure (6 h). Treatment with H_2_O_2_ increased ROS production by 108.9% when compared to control. However, after treatment with FMU200 at 1 µM, ROS production was decreased by 51.08%, while FMU200 at 0.1 µM decreased ROS production by 38.73%. There was no significant difference between FMU200 (1 µM), NAC (5 mM), and the control group, indicating a possible antioxidant effect of FMU200 (Figure 4C).

### 2.3. JNK Inhibition by FMU200

**Figure 5:**
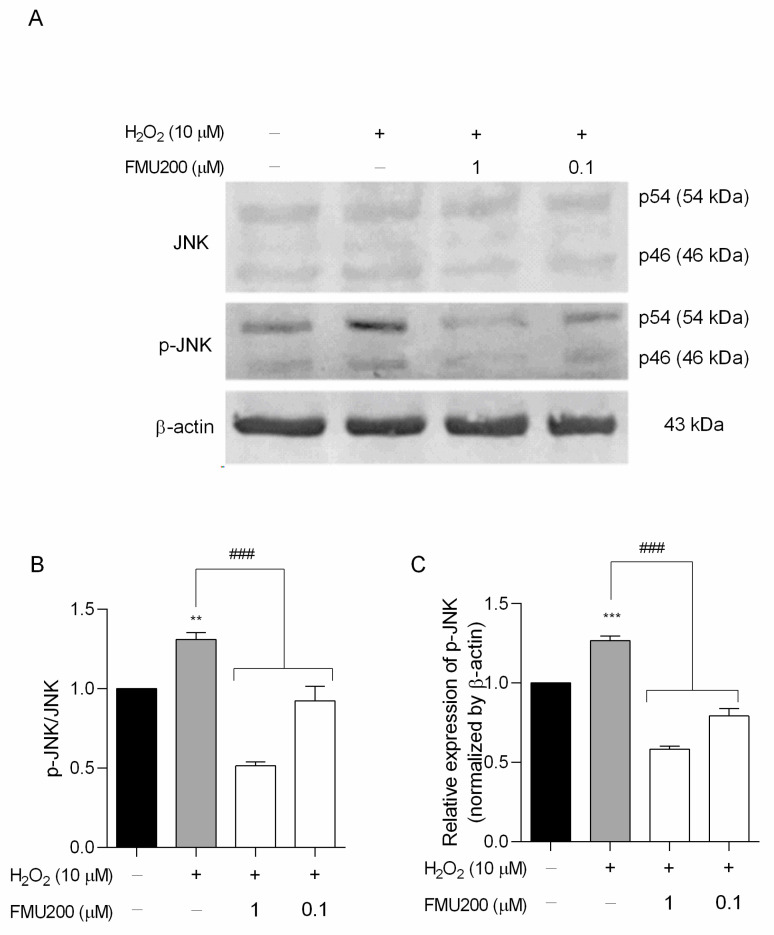
FMU200 inhibited JNK phosphorylation in SH-SY5Y H_2_O_2_-stimulated cells. (**A**) Representative Western blots of total and phosphorylated JNK (p-JNK) showing both JNK isoforms (p46 and p54) and β-actin; (**B**) densitometry ratios of p-JNK to total JNK; (**C**) densitometry for p-JNK protein levels normalized to β-actin. *** *p* < 0.001; ** *p* < 0.01 (vs. control); ### *p* < 0.001 (vs. H_2_O_2_).

The IC_50_ value for JNK3 of FMU200 was previously determined by ELISA assay [21,37]. The authors performed a screening among 410 kinases to investigate the selectivity profile of FMU200 among the kinome and revealed that, in addition to JNK, only three other kinases were inhibited by FMU200 (CK1δ, MAPKAPK2, and TIE2), and the covalent bond to Cys154 of JNK3 was confirmed by liquid chromatography−mass spectrometry (LC−MS). The relevance of covalent binding to Cys154 was previously reported with another potent JNK3 inhibitor (JNK-IN-7) [20]. However, additional virtual simulations suggest that FMU200 may form hydrogen bonds to residues Met149, Asn152, and Gln155, as shown in Figure S1. It was reported that indenoquinoxaline-derived JNK inhibitors, such as IQ-3, were H-bonded mainly with Asn152, Gln155, or Met149 residues of JNK3 indicating that these residues play an important role in enzyme binding activity and selectivity. In this case, it was demonstrated that at 0.1 µM, FMU200 inhibits JNK2 by 73% and JNK3 by 80%, while at 0.5 µM, the residual activity of JNK2 and JNK3 are 5% and 4%, respectively [21]. The representative data acquired via Western blot of JNK inhibition are shown in Figure 5A. Our results indicate that a pretreatment (1 h) with FMU200 (at 1 µM or 0.1 µM) followed a 24 h treatment with H_2_O_2_ decreased the phosphorylated JNK (p-JNK) to total JNK (p-JNK/JNK) ratio by 60.75% and 29.49%, respectively (*p* < 0.001) (Figure 5B), and downregulated p-JNK expression by 54.12% and 37.5%, respectively (*p* < 0.001) if compared to cells treated with H_2_O_2_-only (Figure 5C), confirming the inhibitory activity of FMU200.

### 2.4. Anti-Inflammatory Effect

**Figure 6:**
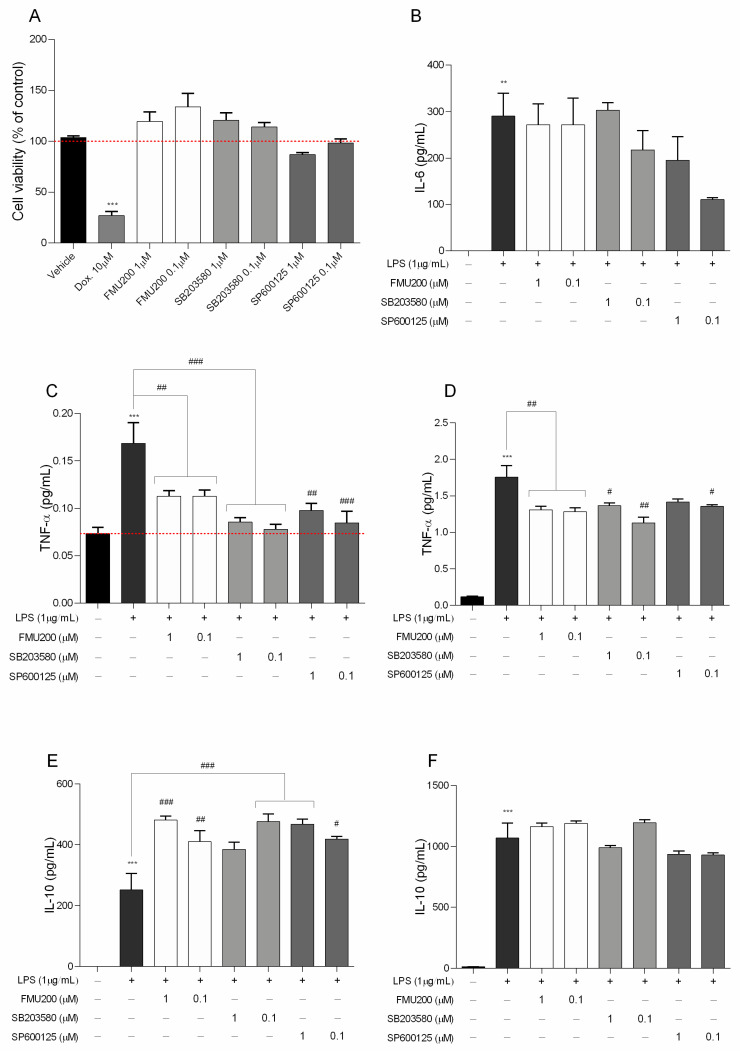
(**A**) Cytotoxicity induced by FMU200 on RAW264.7 cell viability after a 24 h incubation period; negative control (untreated cells) was considered to be 100% viable and is represented by the red dashed line. Doxorubicin was used as positive control; DMSO 0.1% was used as vehicle; (**B**) IL-6 was determined after 12 h of treatment; (**C**) TNF levels after 3 h and (**D**) after 24 h of treatment. IL-10 was evaluated (**E**) after 24 h; and after (**F**) 48 h of treatment. Mean ± SEM of at least three experiments. *** *p* < 0.001; ** *p* < 0.01 (vs. control); ### *p* < 0.001; ## *p* < 0.01; # *p* < 0.05 (vs. LPS or doxorubicin).

As presented in Figure 2A, FMU200 showed moderate cytotoxicity at 10 µM in SH-SY5Y cells. As a result, we determined the cytotoxic effect in RAW264.7 cells at lower concentrations (Figure 6A). No significant reduction in cell viability was found compared with control.

After determining the effects of FMU200 on cell viability ROS production and mitochondrial function, we evaluated proinflammatory cytokines, such as IL-6 and TNF-α, which are thought to be involved in mediating neuroinflammation and inducing neuronal death in various neurodegenerative diseases [38,39]. Additionally, JNK and ROS are also associated with pro-inflammatory events [40].

In the present study, an inflammatory model was successfully established using LPS-stimulated RAW264.7 cells, and therefore, we investigated the effect of FMU200 on the levels of IL-10, IL-6, and TNF-α in LPS-stimulated RAW264.7 cells. TNF-α is the earliest endogenous mediator of an inflammatory reaction, and IL-6 is a major pro-inflammatory cytokine that plays an important role in the acute-phase response of inflammation [41]. Both cytokines can be used as markers of neuroinflammation [42]. To detect its effect over pro-inflammatory cytokines, we pretreated RAW264.7 cells with FMU200 for 1 h, and then exposed these cells to LPS (1 µg/mL) for different time period. IL-6, a pro-inflammatory cytokine was determined after 12 h of co-treatment with LPS and FMU200 at different concentrations. It was revealed that FMU200 did not influence IL-6 levels (Figure 6B). The results demonstrated that after 3 h of LPS stimulation of RAW264.7 cells, treatment with FMU200 at 1 µM and 0.1 µM reduced TNF-α levels by 32.73% and 33%, respectively (Figure 6C). After 24 h, FMU200 at 1 µM and 0.1 µM decreased TNF-α levels by 25.56% and 27.05%, respectively (Figure 6D). Additionally, we evaluated levels of IL-10, an anti-inflammatory cytokine. Since the secretion of IL-10 is delayed and always follows that of proinflammatory factors with a latency period [43], we collected the supernatant after 24 and 48 h. After 24 h, FMU200 at 1 µM and 0.1 µM increases IL-10 levels (91.37%, *p* < 0.01 and 63.05%) (Figure 6E), but after 48 h, FMU200 at 1 µM and 0.1 µM appears to promote a discrete increase (8.89 and 11.32%, respectively) (Figure 6F), but not statistically significant.

## 3. Discussion

The human neuroblastoma cell line SH-SY5Y is widely used as an in vitro experimental model of ND. The undifferentiated SH-SY5Y cells proliferate continuously, express immature neuronal markers, and lack mature neuronal markers [44]. Undifferentiated cells are considered the ones that most resemble immature catecholaminergic neurons [45,46]. Here, we tested whether pretreatment with FMU200 could protect cells against 6-OHDA-induced apoptosis. 6-OHDA is a catecholamine analog that can be formed from dopamine in the presence of Fe^2+^ and H_2_O_2_. It is a substrate for monoamine oxidase (MAO), and competes with dopamine for dopamine β-hydroxylase and COMT reactions, involved in norepinephrine biosynthesis and dopamine degradation, respectively. However, the more cytosolic dopamine, 6-OHDA is more likely to occur, especially if dopamine metabolism is affected by MAO or COMT inhibitors, drugs used in Parkinson’s disease treatment [47].

The main mechanism of neurotoxicity of 6-OHDA relies on two major events. The first one relies on the autoxidation of 6-OHDA where the cell damage is a result of 6-OHDA-derived ROS. Indeed, it is known that 6-OHDA is a highly reactive and oxidizable catecholamine analog that is rapidly and non-enzymatically oxidized by molecular oxygen to form hydrogen peroxide (H_2_O_2_), superoxide anions (O_2_^−^), and hydroxyl radicals (OH^−^) [48]. In all assays, we used 6-OHDA as an apoptosis inducer, and, additionally, we added 0.02% of ascorbic acid to the mixture to avoid rapid 6-OHDA autoxidation [34]. Additionally, it is likely that this effect occurs in vivo since the antioxidants available might be sufficient to prevent rapid oxidation of 6-OHDA, supporting a secondary and alternative route 6-OHDA toxicity: the inhibition of brain mitochondrial complexes I and IV [47].

Several studies indicated that 6-OHDA is a potent inhibitor of complex I and IV in the brain’s mitochondria [47,49,50], inhibiting the mitochondrial respiratory chain complexes mediated by non-radical interactions. Glinka et al. reported that mitochondrial complex I inhibition induced by 6-OHDA is not prevented by antioxidants, and cell death (and therefore neurodegeneration) is caused by ATP depletion [47,51]. It is known that complex I is the main entry point of electrons into the respiratory chain and its inhibition results in the blockade of most of the oxidative metabolic reactions within mitochondria [52], providing support for the theory. Deficiency in complex I of the ETC has been described in PD [53,54] and AD patients [55,56], while decreased complex IV activity and ATP production [57,58] were reported in animal models of AD. In other words, in neurons, one of the 6-OHDA effects is complex I and IV inhibition, causing ATP levels to decrease, facilitating apoptosis. In mice hepatocytes, acetaminophen decreased ATP levels (similarly to 6-OHDA in neurons), while pretreatment with SP600125 prevented a decline in ATP levels, suggesting that JNK translocates to mitochondria and inhibits mitochondrial bioenergetics (at least in part) by triggering mitochondrial permeability transition [59,60]. A similar effect was observed in isolated brain mitochondria, where JNK directly induced mitochondrial permeability transition [61]. Here, we report that FMU200 reduced 6-OHDA-induced cell death in undifferentiated SH-SY5Y cells after 24 and 48 h treatments. Although our results cannot confirm or discard the possibility, it is reasonable to think that FMU200 (like SP600125 treatment in hepatocytes) could prevent a decrease in ATP levels, contributing to the decreased cell death we observed in our MTT assays. We suggest further analysis in order to confirm (or not) the effect of FMU200 over ATP levels.

However, SH-SY5Y cells can be “oriented” to a variety of mature neuronal phenotypes (cholinergic, adrenergic, or dopaminergic), depending on the culture conditions [45]. One of the most commonly implemented and best-characterized methods for inducing differentiation in SH-SY5Y cells is through the addition of retinoic acid (RA) to the cell culture medium. RA is a derivative of vitamin A known to inhibit cell proliferation and induce cell differentiation [62]. Normally, RA is administered at a concentration of 10 μM for a minimum of 3–5 days in a serum-free or low-serum medium to induce differentiation [45,63,64], although small variations in the media are reported. In this sense, other than non-differentiated cells, differentiated SH-SY5Y cells become morphologically more similar to primary neurons with long processes randomly distributed (neurites). Differentiation of SH-SY5Y cells also induces a decrease in the rate of proliferation [44]. The differentiation method selected for in vitro experiments must be determined by the desired phenotype after differentiation. In response to RA treatment, SH-SY5Y cells differentiate mainly into a cholinergic neuron phenotype, as evidenced by the increased expression of choline acetyltransferase (ChAT) and vesicular monoamine transporter (VMAT) activity [46,65]. After differentiation, cells begin to upregulate genes involved in antioxidant defense. This modified gene expression profile directly reflects the cells’ ability to recover from the oxidative stress caused by 6-OHDA [63]. To confirm this greater resistance, a second positive control with doxorubicin (10 μM) was included. When interleaved with the DNA, doxorubicin induces the breaking of the double-strand and, therefore, cell death. In addition, it can inhibit the enzyme topoisomerase II [66,67]. According to our results, FMU200 promoted a neuroprotective effect in RA-differentiated cells after 24 h, but the most prominent effect was observed after 48 h.

After confirming the neuroprotective effect of FMU200 in both undifferentiated and RA-differentiated SH-SY5Y cells, we aimed to understand the mechanism of action of FMU200 and the nature of the effects of FMU200 over different events related to JNK signaling and ROS production that usually precedes apoptosis. It is important to note that in subsequent tests (intracellular levels of ROS and the potential of mitochondrial membrane (∆Ψm)), we used undifferentiated SH-SY5Y cells stimulated by H_2_O_2_. We opted for using undifferentiated cells as our experimental model because the differentiation process promotes a series of modifications that could mask or interfere with our results. RA treatment has been shown to promote the survival of SH-SY5Y cells by activating the phosphatidylinositol 3-kinase/Akt signaling pathway and by positive regulation of the anti-apoptotic Bcl-2 protein [68,69]. In addition, some studies show that RA-differentiated cells are less vulnerable than undifferentiated cells to common agents used to induce cell death including 6-OHDA, 1-methyl-4-phenyl-1,2,3, 6-tetrahydropyridine (MPTP), or its metabolite, ion 1-methyl-4-phenyl-pyridinium (MPP^+^) than undifferentiated cells [63]. Additionally, we evaluated the effect of FMU200 over H_2_O_2_-induced cell injury in the following assays because (a) H_2_O_2_ is the most stable ROS, (b) it transits through cell membranes easily, (c) one of the subproducts of 6-OHDA degradation is H_2_O_2_ [34,47,48], (d) in N18 cells, the damage to cell structure and function induced by 6-OHDA and H_2_O_2_ was similar [70], (e) H_2_O_2_ acts as both extracellular and intracellular messenger [71,72,73] and; (f) the JNK pathway plays a pivotal role in cell death of several cell types and the activation of JNK3 appears to be essential for the pathophysiology of many neurodegenerative diseases, and H_2_O_2_ is widely used as general oxidative stress in vitro model of neurodegenerative diseases [74]. In this case, previous evidence suggested that FMU200, a tetrasubstituted imidazole, forms a covalent bond with JNK3, inhibiting its phosphorylation and downstream activation. Our Western blot analysis is consistent with Muth et al., 2016, indicating a downregulation in p-JNK. In this case, our study supports evidence from previous observations that pointed to a decrease in JNK3 activity. JNK is widely associated with cell death and participates in both extrinsic and intrinsic pathways. In the intrinsic pathway, JNK phosphorylates transcription factors inducing the expression of pro-apoptotic proteins and decreases the expression of anti-apoptotic proteins. The major JNK target is the transcription factor AP-1, which is a complex formed by members of Jun, Fos, ATF, and MAF protein families. JNK phosphorylates ATF2 at the NH_2_-terminal activation domain on Thr69 and Thr71 residues, increasing ATF2 transcriptional activity. However, JNK mediates apoptosis not only through its effects on gene transcription but also through transcriptional-independent mechanisms involved in the intrinsic pathway of cell death. The activation of JNK can simultaneously change the mitochondrial membrane potential (MMP) and the release of cytochrome c, which induces apoptosis via the intrinsic pathway. Since the JNK pathway and ATF2 transcriptional activity can be activated by ROS, it was hypothesized if FMU200 had any effect on ROS production.

Cells constantly generate reactive oxygen species (ROS) during aerobic metabolism. Due to the brain’s high metabolic rate, it consumes almost 25% of the body’s total intake of glucose and 20% of the total oxygen uptake during ATP production. During this process, ROS are also generated as a result of the activity of the electron transport chain (ETC) during oxidative phosphorylation and, as a result, the brain tissue is particularly susceptible to oxidative stress [73,75]. Several events have been associated with neurodegeneration such as synaptic dysfunction, excitotoxicity, and oxidative stress. Indeed, because of its high metabolic rate combined with a limited capacity of cellular regeneration, the brain is particularly sensitive to oxidative damage. The damage caused by reactive oxygen species in specific brain regions was associated with AD, mild cognitive impairment (MCI), Parkinson’s disease (PD), and amyotrophic lateral sclerosis (ALS) [76,77,78,79,80]. In other words, there is evidence linking ROS and the pathophysiology of several neurodegenerative diseases. However, randomized trials evaluated the effects of antioxidants in AD patients, providing conflicting results [81,82]. Higher levels of ROS activate cell death processes [71] and, in this case, antioxidant therapy appears to be insufficient to promote significant improvements, supporting the need for exploring novel targets.

ROS and JNK are highly interconnected, and previous studies reported that treatment with a JNK3 inhibitor (compound 9l or SR-3562) had shown potent inhibition of ROS generation following JNK activation in HeLa cells [10] and INS-1 cells [83]. Here, we demonstrated that FMU200 (at 1 and 0.1 μM) was also able to decrease ROS production, corroborating with studies previously published that evaluated other JNK inhibitors. In addition, SR-3562, like FMU200, prevented ROS formation in a similar way to NAC, a generic antioxidant [10]. In this case, it appears that JNK activation (by H_2_O_2_) induces ROS generation, while the inhibition of JNK by FMU200 decreases ROS production. To provide further support to our results, the radical-trapping antioxidant properties of FMU200 should be evaluated.

**Figure 7:**
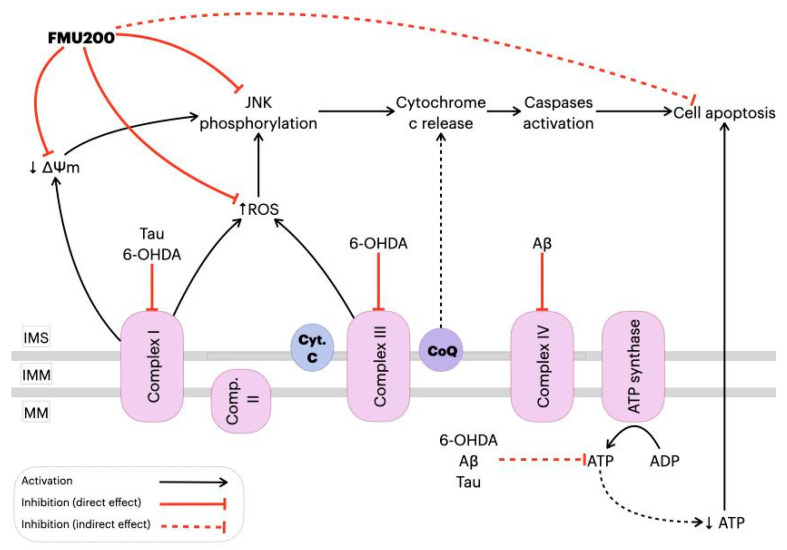
Proposed mechanism of action of FMU200 in mitochondria. Decrease in complex I activity induced by hyper-phosphorylated Tau or 6-OHDA causes an ΔΨm depolarization and, consequently, JNK pathway activation [89,96,97]. In addition to complex I inhibition, 6-OHDA also inhibits complex III [47], while Aβ is reported to inhibit the complex IV [97]. A defective oxidative phosphorylation results in excessive ROS production, which activates the JNK pathway. Additionally, dysfunction of mitochondrial complexes also results in decreased ATP production, which directly leads to apoptosis. However, FMU200 was able to reduce ROS production, prevent ΔΨm depolarization and JNK phosphorylation, demonstrating a protective effect over common AD and PD-related mitochondrial perturbations. ↓: represents decrease; ↑ represents increase. Inner mitochondrial space (IMS); inner mitochondrial membrane (IMM); mitochondrial matrix (MM); cytochrome c (Cyt. C); coenzymeQ (CoQ).

Any increase in mitochondrial ROS production depends on the metabolic state of this organelle and a correlation between mitochondrial membrane potential (ΔΨm) and reactive oxygen species (ROS) production [60,84,85] has been demonstrated. In mitochondrial disorders, decreased ΔΨm and activity of the respiratory chain are observed with a simultaneous increase in ROS production [86,87]. Additionally, ΔΨm depolarization is generally correlated to neuronal death [54,88]. Mitochondrial depolarization induced by JNK was evaluated in Huh7 and HepG2 cells [89]. Heslop and colleagues demonstrated that mitochondrial dysfunction is mediated by JNK activation, while the JNK inhibition by JNK inhibitor VIII and SP600125 prevented mitochondrial dysfunction and blocked JNK translocation to the mitochondria. By preventing the JNK translocation to the outer mitochondrial membrane, a decrease in ROS production [90] was observed. In human melanoma cells, JNK activation was necessary for ΔΨm change and cell apoptosis [91] and treatment with SP600125 prevented both the loss of ΔΨm and the increase in apoptosis by inhibiting JNK activation in different cell types [92,93,94,95]. One possible explanation for these findings is that JNK plays a significant role in apoptosis via the intrinsic pathway (also known as the ‘mitochondrial pathway’), which is activated by extracellular or intracellular perturbations usually found in AD, such as oxidative stress. In response to a deleterious stimulus (such as ROS), JNK phosphorylates 14-3-3 protein and induces the translocation of pro-apoptotic proteins (Bax and Bad) from the cytoplasm to the mitochondria, the major source of ROS in cells. However, it was reported that JNK can directly phosphorylate Bad, Bim, and Bid inducing their pro-apoptotic activity while inhibiting anti-apoptotic proteins. Once translocated to the mitochondria, JNK increases ROS formation in 80%, especially by complex I [10]. In the present study, FMU200 attenuated H_2_O_2_-induced production of intracellular ROS and inhibited H_2_O_2_-induced depolarization of ΔΨm, which are important molecular markers for reflecting the mitochondria oxidative stress status. These results connect the JNK pathway directly with mitochondrial-dependent apoptosis, suggesting that mitochondria are an important target organelle of FMU200 and may be essential for its neuroprotective action (Figure 7).

Despite its major contribution to the pathophysiology of neurodegenerative diseases through pro-apoptotic signals, JNK can also promote pro-inflammatory effects [98]. In AD, for example, neuroinflammation relies on an innate immune response mediated by microglia [99]. After a stimulus, the microglia produce several inflammatory mediators, such as IL-1β, IL-6, TNF-α, prostaglandin E2 (PGE2), nitric oxide (NO), brain-derived neurotrophic factor (BDNF), which can activate the JNK pathway. The major contribution of JNK to neuroinflammation is via its transcription factor, AP-1, which regulates proinflammatory genes such as *COX2*, *NOS2*, *TNF-*α, *CCL2*, and *VCAM-1* [100], and evidence suggests that ROS production induced by TNF-α is JNK-dependent [101,102]. In this case, we identified that FMU200 decreased TNF-α release in RAW264.7 cells after a 3 h treatment, but there was a smaller reduction in TNF-α after 24 h. In general, authors report a decrease in proinflammatory cytokines along with an increase in anti-inflammatory cytokines in LPS-induced RAW264.7 cells treated with SP600125 [103,104,105,106]. The cytotoxicity of different concentrations of LPS was evaluated in RAW264.7 cells by Tong et al. According to their results, LPS at a maximum concentration of 1.25 µg/mL was not cytotoxic, (which provides support for the LPS concentration we used) and cytokine release (TNF-α and IL-6) was dose-dependent to LPS concentration. In this case, it should be mentioned that in our study cells were pretreated with LPS at higher concentrations compared to other studies (1 µg/mL vs. 0.5 µg/mL [103,106] vs. 0.1 µg/mL [104,105]. Furthermore, in these studies, LPS-stimulated RAW264.7 cells were exposed to concentrations of SP600125 of 10 µM [103,106] and 20 µM [104]. Since data from our initial screening with SH-SY5Y (Figure 2A) indicated increased cytotoxicity of SP60125 at 10 µM, we cannot perform a direct comparison. It is important to bear in mind the possible bias in these conflicting responses. On the other hand, other previous work reported that RAW264.7 cells treated with SP600125 reduced c-Jun activation but did not impact IL-1β, IL-6, and TNF-α at mRNA and protein levels [107].

In addition to TNF-α, IL-6 levels were also evaluated. Apparently, FMU200 has no impact over IL-6. Although IL-6 is understood as a pro-inflammatory interleukin, it is a pleiotropic cytokine with a multitude of functions [108]. The authors showed that the cytokine signaling suppressor 3 (SOCS-3) is a key regulator for the pro-inflammatory action of IL-6 and anti-inflammatory of IL-10, and, in the absence of SOCS-3, IL-6 induces an anti-inflammatory response [109]. In fact, a higher amount of SOCS-3 mRNA was found in post-mortem analysis of AD patients [110]. Furthermore, a 20 year cohort observed a correlation of cognitive impairment and higher or increasing levels of IL-6 over the years [111]. On the other hand, the hypothesis that IL-6 attenuates the neurotoxic effects of NMDA on cholinergic neurons has been discussed for almost 30 years [112,113,114,115]. Excessive stimulation caused by glutamate in NMDA receptors causes a phenomenon known as “excitotoxicity” and induces cell death through JNK activation [116,117]. It is noteworthy that meanwhile, one of the drugs used for treating AD is an NMDA receptor antagonist [118] and that treatment with JNK inhibitors such as D-JNKI1, SP600125 or TAT-JNK-III protects against glutamate excitotoxicity and cell death in vivo and in vitro [119,120,121]. In addition, chronic exposure to exogenous IL-6 prevented neuronal death and an increase in NMDA-induced caspase-3 activity. Both AG490 (JAK2 inhibitor) and PD98059 (ERK inhibitor) blocked the protection of IL-6 against a decrease in neuronal vitality induced by NMDA and increased activation of caspase-3 [122,123]. Still, recently, it was demonstrated that the inhibition of IL-6 could contribute to the worsening of depressive disorders [124] which are more prevalent in the elderly population and, even more so, in the elderly population with some neurodegenerative disease [125,126,127]. In this sense, it can be inferred that the neuroprotective effect of IL-6 depends on the concentration of IL-6 and the degree of neuronal damage. It is hypothesized that total blockage of the IL-6 production is not beneficial in the treatment of neurodegenerative diseases. In this case, an IL-6 modulation through JNK inhibition is preferable. Such a modulatory effect could be achieved through treatment with FMU200, since treatment with SR3306, a selective inhibitor of JNK2/3, reduced the expression of SOCS-3 in in vivo models [128]. The fact that in the present study IL-6 levels were not affected by FMU200 can therefore be understood as an ambiguous result.

Levels of a second interleukin were also evaluated. IL-10 is known to inhibit secretion of pro-inflammatory cytokines (IL-1α, IL-1β, IL-6, TNF-α) induced by LPS or IFN-γ [129,130]. In the present study, pretreatment with FMU200 (1 and 0.1 μM) increased levels of IL-10 after 24 h compared to the group treated with LPS only, while after 48 h no effect on IL-10 levels was observed. RAW264.7 cells stimulated with 1 µg/mL LPS (the same concentration we used), SP600125 at 2 µM apparently had a minimal effect over IL-10 [131]. Despite IL-10 being known to inhibit pro-inflammatory cytokines, an increase in *IL10* expression in several animal models of AD provoked a decrease in Aβ phagocytosis by microglia and exacerbated Aβ deposits, leading to cognitive impairment. On the other hand, blocking *IL-10* promotes a reduction in IL-10/STAT3 signaling, and seems to increase microglial phagocytic activity [132,133]. It is important to emphasize that higher IL-10 levels were found in patients with AD [134,135,136,137].

Neurons, microglial cells and macrophages, for example, are also known to express TLR receptors (especially TLR2, 4 and 9) and these receptors are overexpressed in patients with Alzheimer’s or Parkinson’s diseases, and in various experimental models of these diseases [138]. In addition, there is a consensus that the activation of TLR receptors triggers the JNK pathway. Despite the deleterious effects of TLR receptor activation, it is known that IL-10 synthesis depends, in part, on TLR activation. It has been shown that TLR stimulation leads to MAPK activation, which then modulates IL-10 production. On the other hand, the inhibition of ERK, p38 or JNK in LPS-stimulated macrophages led to a significant reduction in IL-10 [138,139,140,141,142,143,144]. In addition, transcription factors activated by JNK such as ATF-1, MAF, NF-kβ (p65), JUN, CREB, have been described to regulate IL-10 expression [145]. Such observations could partially explain the results we observed after 48 h, since FMU200, as a JNK inhibitor, may be negatively modulating IL-10 expression. Finally, it is evident that there is a complexity in the regulation of cytokines through positive and negative feedback cycles and that strict control is essential to achieve a balance between an effective immune response and immunopathology. Collectively, these results suggest that the rebalancing of innate cerebral immunity and the promotion of “beneficial neuroinflammation” may be more effective than a generalized anti-inflammatory therapy for AD. Although the classification of cytokines as “pro” or “anti-inflammatory” is widely adopted in the literature and interesting from a didactic point of view, it is a reductionist characterization and should be avoided since the beneficial or harmful actions of IL-10 and IL-6 depend on a broader context.

Despite the promising potential of FMU200, there are two major limitations in this study that could be addressed in future research. First, the primary focus of our study was to unravel the effect of FMU200 over JNK3-related apoptosis in in vitro models of neurodegenerative diseases and, therefore, the mechanisms underlying the anti-inflammatory effect of FMU200 were not totally explored. Thus, the evaluation of inflammatory mediators such as iNOS and COX-2, as well as, growth factors (i.e., neurotrophins), transcription factors (i.e., Nrf2), and cell receptors (i.e., TLR4) would be of extreme value to fully elucidate the anti-inflammatory mechanisms of FMU200, especially based on co-culturing systems with neuron and microglia-derived cells, for example. Second, although FMU200 is highly selective to JNK3, some of the reported effects might also be due to the inhibition of off-target kinases. The clinical implications of this study are unclear at this point, but based on our observations, we highly suggest further analysis with FMU200 in order to fully elucidate its mechanism of action and further explore its beneficial effects.

## 4. Materials and Methods

### 4.1. Cell Lines and Reagents

Dulbecco’s modified Eagle medium (DMEM) (D5523), F12 (N6760), heat-inactivated fetal bovine serum (FBS) (F4135), 6-hydroxydopamine hydrobromide (6-OHDA) (162957), 3-[4,5-dimethylthiazol-2]-2,5 diphenyltetrazolium bromide (MTT) (M5655), penicillin (P3032), streptomycin (S9137), LPS (from *Escherichia coli*, O111:B4, L2630), trypsin-EDTA (T4049), 2′,7′-Dichlorofluorescin diacetate (DCFDA) (D6883), Protease Inhibitor Cocktail (P8340), and 3-3’-diaminobenzidine (DAB) (D8001) were purchased from Sigma-Aldrich™ (St. Louis, MO, USA). DMEM (1200-058) used to culture RAW264.7 cell line and enzyme-linked immunosorbent assay (ELISA) kits for TNF-α, IL-6, and IL-10 were obtained from Gibco^®^, Invitrogen Life Science Technologies (Grand Island, NY, USA). All-trans-retinoic acid (ATRA) (SC200898) and the primary antibodies for total JNK (D-2), phospho-JNK (Thr183/Tyr185) (G-7), and β-actin (C-4) were purchased from Santa Cruz Biotechnology, (Dallas, TX, USA). Spectrophotometer-based analyses were performed using SpectraMax^®^ i3 (Molecular Devices, San Jose, CA, USA). 5,5,6,6-tetrachloro-1,1,3,3-tetraethylbenzimidazolylcarbocyanine iodide (JC-1) staining is from Molecular Probes (Eugene, OR, USA). PE Annexin V Apoptosis Detection Kit I was purchased from BD Biosciences (USA). Secondary antibodies for anti-mouse IgG, HRP-linked, (7076) and anti-rabbit IgG, HRP-linked (7074) were bought from Cell Signaling Technology (Danvers, MA, USA). SH-SY5Y (ATCC© CRL-2266™) and RAW264.7 cell line (ATCC© TIB-71™) was acquired from American Type Culture Collection (ATCC). FMU200 and the MAPK inhibitors (SP600125 and SB203580) were synthesized by research of Prof. Dr. Stefan Laufer with a high purity grade (≥95%).

### 4.2. Cell Culture Methods

#### 4.2.1. SH-SY5Y Cell Line

Undifferentiated human SH-SY5Y neuroblastoma cells were cultured in DMEM mixed with F12 (1:1) and supplemented with 10% (*v*/*v*) FBS and 1% streptomycin/penicillin under controlled conditions in a 95% humidified atmosphere, at 37 °C and 5% CO_2_. The culture medium was replaced every two days until the cells reached confluence 4–5 days after the initial seeding. For subculture, SH-SY5Y cells were dissociated with trypsin-EDTA (0.25%), split into a 1:3 ratio. Cells were grown to 80% confluence before treatment. Culture conditions were performed according to ATCC recommendations.

#### 4.2.2. SH-SY5Y Differentiation Protocol

To differentiate the SH-SY5Y cells, we adapted a previously described protocol and exposed cells to 10 μM of ATRA for 10 days [45].

#### 4.2.3. RAW264.7 Cell Line

RAW264.7 cells were cultured in DMEM supplemented with 10% (*v*/*v*) of FBS and 1% of streptomycin/penicillin. The medium was replaced every 2 to 3 days. Subculturing was carried out with a cell scraper at a 1:4 split ratio. All procedures were made following ATCC recommendations.

### 4.3. Determination of Cell Viability and Neuroprotection Potential by MTT Assay

#### 4.3.1. MTT Assay and Cytotoxicity of FMU200

Cell viability was assessed using the colorimetric MTT assay. SH-SY5Y (differentiated and undifferentiated) and RAW264.7 cells were seeded in 96-well dishes and left overnight in the incubator for proper attachment. Cells were exposed to different concentrations (10–0.1 μM) of FMU200 for 24 or 48 h. At the end of the incubation period, MTT reagent was applied to each well at a final concentration of 5 mg/mL and the plate was placed in a humidified incubator at 37 °C with 5% of CO_2_ for a further 3 h period. Formazan salts were dissolved in DMSO and the colorimetric determination of the reduction of MTT was determined at 570 nm wavelength using the spectrophotometer SpectraMax^®^ i3. Control cells treated with maintenance media were considered to be 100% viable.

#### 4.3.2. Neuroprotection Potential

To investigate the neuroprotective potential of the compound, SH-SY5Y (differentiated and undifferentiated) cells were seeded at a density of 2 × 10^4^ per well in a 96-well dish and left in the incubator overnight. Next, the medium was replaced with different concentrations (1 or 0.1 μM) of FMU200 for 30 min before adding 100 μM of 6-OHDA stabilized with 0.02% of ascorbic acid to avoid auto-oxidation of 6-OHDA. After 24 or 48 h, the treatment was removed, and MTT (5 mg/mL) was added for 3 h. Following the MTT removal, DMSO was used to dissolve the formazan salts and the OD was evaluated at 570 nm using a spectrophotometer (SpectraMax^®^).

### 4.4. Apoptosis Assay by Flow Cytometry

PE Annexin V versus 7-aminoactinomycin D (7-AAD) staining was performed and analyzed by flow cytometry. Briefly, cells were plated into 6-well culture dishes (3 × 10^5^ cells/well) and left overnight for attachment. The next day, cells were pretreated for 1 h with different concentrations of FMU200. Following the 1 h pretreatment, apoptosis was induced with H_2_O_2_ for an additional 5 h. After 6 h of incubation, cells were harvested, washed with cold PBS 1X, and suspended in 1× binding buffer at a concentration of 1 × 10^6^ cells/mL. Then, 100µL of the cell suspension were added to a tube, treated with 5 µL of PE Annexin V and 5 µL of 7-AAD, and incubated for 15 min at room temperature in the dark, according to the manufacturer’s instructions. The fluorescence was immediately determined by a flow cytometer (Accuri C6, BD Biosciences, San Jose, CA, USA) using FL-2 and FL-3 filters.

### 4.5. Mitochondrial Membrane Potential (MMP) Assay

Cells were seeded in 96-well plates and left in the incubator overnight. Next, SH-SY5Y cells were treated with H_2_O_2_ (100 μM) for 6 h, in the absence or presence of FMU200 (0.1 or 1 μM). After the appropriate period of exposure, cells were washed with PBS 1X and incubated with JC-1 at 37 °C for 30 min. Then, the reagent was gently removed, and cells were washed with PBS 1X. 100 μL/well of PBS 1X was added and the fluorescence was measured at 540/570 nm (red fluorescence) and 485/535  nm (green fluorescence) using a fluorescence microplate reader SpectraMax^®^ i3. Mitochondrial membrane potential was estimated by measuring the fluorescence of free JC-1 monomers (green) and JC-1 aggregates in mitochondria (red) and the results were expressed as the ratio of the aggregates/monomers of JC-1 in the percentage of control. Mitochondrial depolarization was indicated by a decrease in the polymer/monomer fluorescence intensity ratio. We included carbonyl cyanide *m*-chlorophenyl hydrazone (CCCP) as a positive control.

### 4.6. ROS Production

ROS production was measured by fluorogenic dye H_2_-DCFDA, which is oxidized by intracellular ROS. Cells seeded in 96-well plates were treated with FMU200 at different concentrations (0.1 or 1 μM) and 10 μM H_2_O_2_ for 6 h. Following treatment, cells were washed with PBS (1X) and incubated with carboxyH2DCFDA for 1 h at 37 °C. Next, the fluorescent compound was detected by a fluorescence microplate reader with excitation and emission of 495 and 529 nm, respectively.

### 4.7. Western Blot

SH-SY5Y cells were pretreated (1 h) with FMU200 at 0.1 or 1 μM before H_2_O_2_ exposure (24 h). Cells were washed with PBS and then harvested with radioimmunoprecipitation assay buffer (RIPA) [150 mM NaCl, 50 mM Tris-HCl, 0.5% NP-40, 0.1% sodium dodecyl sulfate (SDS), 1 mM EDTA, pH 7.4] supplemented with protease inhibitors and phosphatase inhibitors (NaF and Na_3_VO_4_). Cells were incubated with lysis buffer at 4 °C for 30 min while rocking gently. Cells were scraped from the culture surface and transferred to a microcentrifuge tube. The cell lysate was centrifuged at 14,000× *g* for 15 min to remove cellular debris. Protein concentrations of total cell lysates were measured by Lowry assay [146]. For Western blot analysis, proteins were resolved by SDS-PAGE, and transferred to nitrocellulose membranes. Membranes were incubated with TBST buffer (1X TBS, 0.1% Tween-20 with 5% *w/v* non-fat dry milk [NFM] or 5% bovine serum albumin [BSA], pH 7.4) for 2 h. For phospho-Western blots, membranes were blocked with TBST buffer containing 5% BSA (BSA/TBST buffer) rather than non-fat milk (NFM/TBST buffer). The membranes were incubated with primary antibodies specific for total JNK, phospho-JNK (Thr183/Tyr185), or β-actin at dilutions of 1:300 in BSA/TBST or NFM/TBST buffer. Membranes were washed three times for 5 min in 1X TBST. Membranes were incubated with secondary antibodies in the BSA/TBST or NFM/TBST buffer at 1:3000 for HRP-conjugated antibody (anti-mouse IgG, HRP-linked) and the reaction was revealed with DAB [147]. Densitometry analysis was performed with the ImageJ gel analysis plug-in as described elsewhere [148].

### 4.8. Cytokine Determination in RAW264.7 Cell Line

The evaluation of the anti-inflammatory potential of FMU200 was performed as previously described [149]. In summary, RAW264.7 cells were seeded at a density of 5 × 10^5^ cells/well in a 24-well plate. After adherence time, cells were pretreated with FMU200 for 1 h before LPS (1 μg/mL) was added. The supernatant was collected at different times. To evaluate TNF-α release, samples of supernatant were collected after 3 and 24 h of treatment. For IL-6 analysis, samples were collected after 12 h of treatment. For IL-10, samples were analyzed after 24 and 48 h of treatment. All samples were frozen at −80 °C until the analysis. The ELISA assay was performed according to the manufacturer’s instructions. The absorbances were measured at 450 and 570 nm using a spectrophotometer (SpectraMax^®^ i3). Values of 570 nm were subtracted from those of 450 nm to remove background interference. TNF-α, IL-6 and IL-10 standard curves were used to quantify the release from each cytokine.

### 4.9. Statistical Analysis

The statistical analysis was performed on GraphPad Prism 6.0 software using ANOVA. The results are expressed in mean ± standard error of the mean (SEM). A *p* < 0.05 was considered statistically significant.

## 5. Conclusions

**Figure 8:**
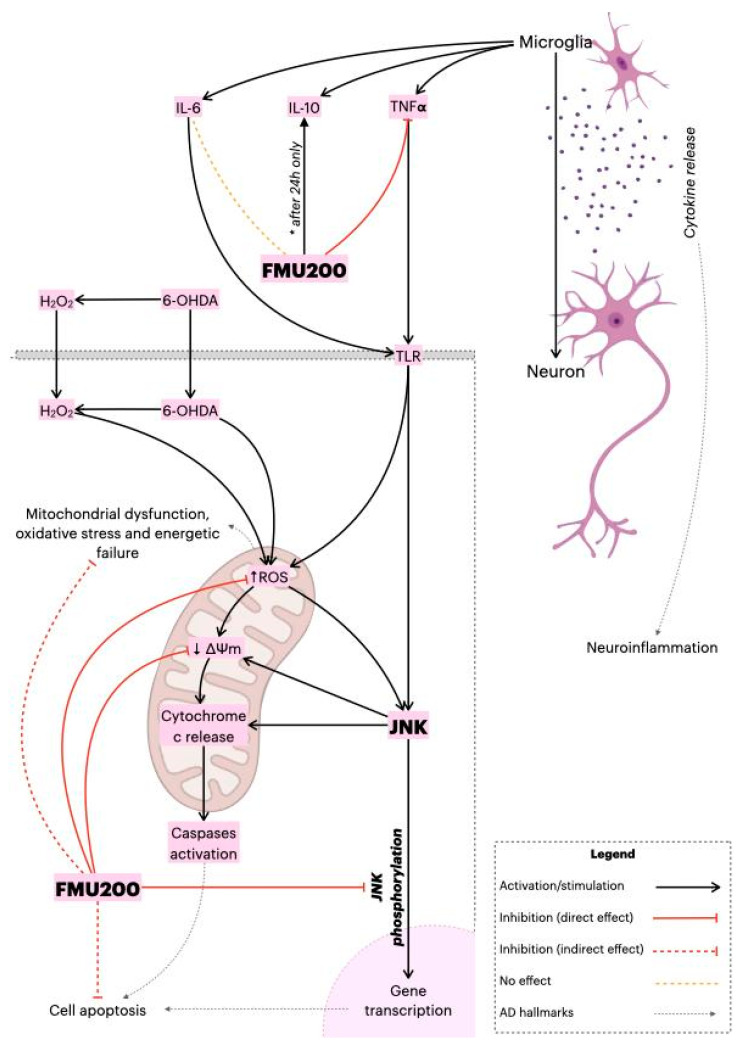
Proposed overall mechanism of action of FMU200. FMU200 blocked the phosphorylation of JNK and, therefore, inhibited apoptosis. Regarding the mitochondria, the direct effect of FMU200 was to reduce ROS production and prevent ΔΨm depolarization. As an indirect result, FMU200 contributed to reduce mitochondrial dysfunction, oxidative stress, and (possibly) to energetic failure. In a microglia model, FMU200 was able to reduce TNF-α production, and induce IL-10 (after 24 h), which contributes to modulate the neuroinflammation.

The present study supports our hypothesis that the neuroprotective effects of FMU200 were mediated by mitochondrial protection, a reduction in oxidative stress conditions, inflammation, apoptosis, and inhibition of the JNK pathway as proposed in Figure 8. It is possible that the inhibition of JNK by FMU200 prevents apoptotic death by downregulating p-JNK but also decreasing mitochondrial disruption, as shown in Figure 7. In conclusion, considering the role of JNK3 in ND’s, the limited number of pharmacologic therapies in AD, the use of kinase inhibitors in treating other diseases, and the results of our report, FMU200 demonstrated to be a promising molecule and should be considered in further and more complex researches.

## Data Availability

Not applicable.

## Supplementary Materials

The following are available online at https://www.mdpi.com/article/10.3390/ijms22073701/s1.
