# Supplementary material for: A Highly Selective In Vitro JNK3 Inhibitor, FMU200, Restores Mitochondrial Membrane Potential and Reduces Oxidative Stress and Apoptosis in SH-SY5Y Cells
Source: Int J Mol Sci. 2021 Apr 2;22(7):3701. doi: 10.3390/ijms22073701 (PMC8037381; doi:10.3390/ijms22073701)
Supplement: Supplementary file 1 [file ijms-22-03701-s001.pdf]

Supplementary material:

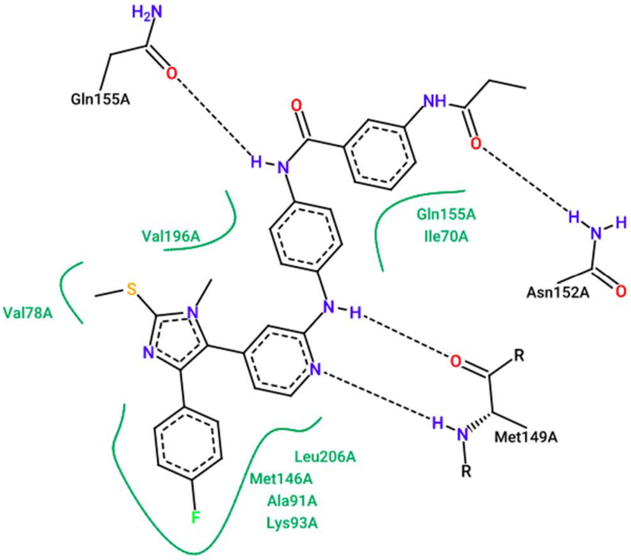

Figure S1. 2D protein-ligand interaction plot for 1PMN, emphasizing the interactions between the ligand (FMU200) and the residue Met149, Asn152, and Gln155. The hydrogen bonds (at atom level) are shown in dashed lines. Amino acids that do not interact with the ligand via hydrogen bond are shown in green. Water molecules are not shown. Plot automatically generated by ProteinsPlus using the PoseView tool based on the 2Ddraw library.
